# Rehabilitation following rotator cuff repair: A nested qualitative study exploring the perceptions and experiences of participants in a randomised controlled trial

**DOI:** 10.1177/0269215520984025

**Published:** 2020-12-27

**Authors:** Gareth Stephens, Chris Littlewood, Nadine E Foster, Lisa Dikomitis

**Affiliations:** 1 The Royal Orthopaedic Hospital NHS Foundation Trust, Birmingham, UK; 2 Department of Health Professions, Faculty of Health, Psychology and Social Care, Manchester Metropolitan University, Manchester, UK; 3 Primary Care Centre Versus Arthritis, School of Primary, Community and Social Care, Keele University, Staffordshire, UK; 4 School of Medicine, Keele University, Staffordshire, UK

**Keywords:** Qualitative study, physical therapy, rehabilitation, shoulder pain

## Abstract

**Objective:**

To investigate acceptability, barriers to adherence with the interventions, and which outcome measures best reflect the participants’ rehabilitation goals in a pilot and feasibility randomised controlled trial evaluating early patient-directed rehabilitation and standard rehabilitation, including sling immobilisation for four weeks, following surgical repair of the rotator cuff of the shoulder.

**Design:**

Nested qualitative study.

**Setting:**

Five English National Health Service Hospitals.

**Subjects:**

Nineteen patient participants who had undergone surgical repair of the rotator cuff and 10 healthcare practitioners involved in the trial.

**Method:**

Individual semi-structured interviews. Data were analysed thematically.

**Results:**

Four themes: (1) Preconceptions of early mobilisation; many participants were motivated to enter the trial for the opportunity of removing their sling and getting moving early. (2) Sling use and movement restrictions; for some, sling use for four weeks was unacceptable and contributed to their pain, rather than relieving it. (3) Tensions associated with early mobilisation; clinical tensions regarding early mobilisation and the perceived risk to the surgical repair were apparent. (4) Processes of running the trial; participants found the trial processes to be largely appropriate and acceptable, but withholding the results of the post-operative research ultrasound scan was contentious.

**Conclusion:**

Trial processes were largely acceptable, except for withholding results of the ultrasound scan. For some participants, use of the shoulder sling for a prolonged period after surgery was a reported barrier to standard rehabilitation whereas the concept of early mobilisation contributed tension for some healthcare practitioners due to concern about the effect on the surgical repair.

## Introduction

We conducted a multi-centre pilot and feasibility randomised controlled trial to evaluate the feasibility of a fully powered randomised controlled trial to compare the clinical and cost-effectiveness of early patient-directed rehabilitation with standard rehabilitation, incorporating sling immobilisation for four-weeks following surgical repair of the rotator cuff.^1^ Patients diagnosed with non-traumatic tears of the rotator cuff and listed for surgical repair were recruited from five English National Health Service Hospitals and randomly allocated to early patient-directed rehabilitation or standard rehabilitation. The comprehensive protocol describing the methods and interventions has been published^1^ and the results of the pilot and feasibility randomised controlled trial have been reported in a linked paper.^2^

Alongside the pilot and feasibility randomised controlled trial we conducted this nested qualitative study with the following objectives:

(1) To understand the acceptability of the interventions to participants and healthcare practitioners.
(2) To explore the barriers and enablers to adherence with interventions.
(3) To understand which outcome measure(s) best reflect(s) the participants’ rehabilitation goals.

Effective rehabilitation is considered important to help patients achieve the best clinical and quality of life outcomes. Despite this, there is uncertainty about the optimal approach including when a patient should remove their sling or brace, provided after the surgery to immobilise the shoulder, and when they should begin moving their shoulder.^3–5^

## Methods

A qualitative study was undertaken using semi-structured individual interviews. The comprehensive study protocol has been published previously.^1^ This study is reported in accordance with the consolidated criteria for reporting qualitative research (COREQ)^6^ This study was funded by the National Institute for Health Research (NIHR) Research for Patient Benefit programme (PB-PG-0816-20009). This study was sponsored by Keele University (RG-0038-16-PCHS). A favourable ethical review was granted by the Wales Research Ethics Committee 5 Bangor on 31st July 2018 (18/WA/0242). Recruitment to the pilot and feasibility randomised controlled trial took place between November 2018 and November 2019 and recruitment to this qualitative study took place between those dates.

During the consent process for participation in the randomised controlled trial, patient participants could consent to further contact to discuss participation in this qualitative study. The initial plan was to recruit according to allocated treatment group and the participants’ pain and disability status according to early text message responses. However, given the similarity in early text message responses this was not feasible. When consent to contact was gained, patient participants were contacted by the researcher (GS), to discuss participation and if they wished to participate a participant information sheet and a consent form was posted. On receipt of written informed consent, an interview was scheduled.

Healthcare practitioners involved in the trial, including orthopaedic surgeons, physiotherapists and site-based research staff, including nurses and physiotherapists, were eligible to participate. Healthcare practitioners were informed during site set-up visits where consent to contact, healthcare practitioner participant information sheet and consent forms were provided. Healthcare practitioners, who consented to an initial contact telephone call, were called to discuss participation. On receipt of written informed consent, an interview was scheduled.

The recruitment targets were up to 20 patient participants and up to 10 healthcare practitioners, guided by data saturation. Data saturation was determined by the point at which all themes emerging from the data had been sufficiently represented, and no new themes were being generated.^7^

In-depth semi-structured, telephone and face-to-face interviews were conducted by the researcher (GS) who was unknown to patient participants but known professionally to two healthcare practitioner participants. GS is a male clinical-academic physiotherapist, with experience of conducting semi-structured qualitative interviews. The interviews were guided by topic guides (Supplemental Appendices 1, 2 and 3) developed in collaboration with a patient and public involvement group.^1^ The topic guides covered: (1) the processes of recruitment; (2) the interventions and (3) data collection (text messages, postal questionnaires, ultrasound scan at 12 weeks). The topics were covered flexibly to enable exploration of any new and unanticipated issues. The interviews were audio recorded and transcribed verbatim.

The data were analysed thematically in the context of individual patient journeys using the six-step approach outlined by Braun and Clarke.^8^ Transcripts were read and re-read in their entity by the researcher (GS) and initial codes were generated in the QSR NVIVO 12 software.^9^ These codes and subsequent themes were discussed amongst the wider research team (GS, CL, LD) and the transcripts were read in their entirety by a second researcher (CL) to check that the coding was comprehensive before further discussion and refinement of the ‘thematic map’.

## Results

The first 35 patient participants who consented to contact were called. Eight did not answer three separate telephone calls. Twenty-seven expressed further interest and 19 participants (early patient-directed rehabilitation = 11, standard rehabilitation = 8) returned a completed consent form and participated in a telephone interview. All ten healthcare practitioners (physiotherapists = 6, research staff = 2, surgeons = 2) who were approached returned their consent forms and participated in an interview (telephone = 8, face-to-face = 2). Data saturation was confirmed at this point as no new themes were identified during the final three interviews.

The pilot and feasibility randomised controlled trial recruited participants from five National Health Service hospital sites in England (Tables 1 and 2). The 10 healthcare practitioner interviews lasted a mean duration of 22 minutes (range 12–39), and the patient interviews lasted a mean duration of 25 minutes (range 17–37).

**Table 1.** Characteristics of the patient participants.

Trial number	Gender	Age	Employment status	Randomised controlled trial allocation
214	F	64	Housewife	Early patient-directed rehabilitation
223	F	61	Retired	Early patient-directed rehabilitation
229	M	74	Retired	Early patient-directed rehabilitation
230	M	59	Employed	Standard rehabilitation
233	F	74	Retired	Standard rehabilitation
241	M	77	Retired	Early patient-directed rehabilitation
242	F	71	Retired	Standard rehabilitation
260	F	67	Unemployed	Standard rehabilitation
406	M	63	Employed	Standard rehabilitation
407	M	73	Retired	Standard rehabilitation
437	F	75	Retired	Early patient-directed rehabilitation
449	F	63	Unemployed	Early patient-directed rehabilitation
463	F	67	Retired	Early patient-directed rehabilitation
603	M	74	Retired	Standard rehabilitation
608	M	68	Retired	Early patient-directed rehabilitation
609	M	58	Retired	Early patient-directed rehabilitation
801	M	63	Other (not specified)	Early patient-directed rehabilitation
803	F	57	Employed	Early patient-directed rehabilitation
804	M	72	Retired	Standard rehabilitation

F: female; M: male.

**Table 2.** Characteristics of healthcare practitioner participants.

Trial number	Gender	Role	Role in the trial	Years of Clinical experience
H01	F	Physiotherapist	ID	19
H02	F	Research nurse	Recruitment	9
H03	F	Clinical trials practitioner	Recruitment	13
H04	F	Research Physiotherapist	Recruitment & ID	26
H05	F	Research Physiotherapist	Recruitment & ID	15
H06	M	Surgeon	Surgeon	18
H07	F	Physiotherapist	Recruitment & ID	10
H08	M	Physiotherapist	ID	8
H09	M	Surgeon	Surgeon	21
H10	F	Physiotherapist	ID	15

ID: intervention delivery.

Three themes that focus on the acceptability of the interventions and adherence were identified:

(1) Preconceptions of early mobilisation
(2) Sling use and movement restrictions
(3) Tensions associated with early mobilisation.

A fourth theme covered the appropriateness of outcome measures and evaluation of the randomised controlled trial processes.

The risks and benefits of early patient-directed rehabilitation were discussed consistently. Most patient participants were keen to remove their sling soon after surgery due to the limitations imposed:

*‘. . .I found it quite difficult to, to accept (being told to keep the sling on for four weeks) be-, - simply because of being on my own. I'm right-handed and it was my right shoulder’ [P242]*

Also, the perception that early patient-directed rehabilitation would facilitate the healing process:

*‘. . . if you don’t use it you lose it sort of thing. . .’ [P229]*

*‘. . . If you keep wearing the sling er, the muscles will not be using exercises and so forth. So you might remain like that for some time and it won’t heal. So I wanted it to heal and move. . .’ [P463]*

In one case, the impact of the sling on self-identity appeared to be a barrier to adherence:

*‘. . .I don’t like to look like a wounded bird wandering round the place. . .’ [P608]*

The positive views of early patient-directed rehabilitation were largely shared by the physiotherapists:

*‘I was confident. I was very confident in getting them out the sling. . .’ [H08]*

Preconceptions that early patient-directed rehabilitation could be harmful were apparent but not raised directly. However, some healthcare practitioners reported that some patients who were approached to take part in the trial were fearful of early patient-directed rehabilitation and refused to participate:

*‘I have had one or two that absolutely adamant that they didn’t want to be out of sling but that’s based on previous surgeries mostly. . .’ [H02]*

‘. . .we had recruited one patient, but he’d dropped out of the trial because his brother came and said, “You can’t leave the sling off. You have to wear the sling as the shoulder needed protection”’ [H06]

The preconceptions of healthcare practitioners were more commonly reported as a barrier to recruitment. Reports of surgeons being reluctant to allow their patients to participate were recurrent:

*‘The main problem we had was that we only managed to persuade one of our surgeons to let his patients take part. All the others weren’t interested. . .they were worried taking the sling off would damage the surgery’ [H02]*

Patient participants in the standard rehabilitation group described finding it difficult to comply with wearing the sling for four weeks:

*‘. . .I had no idea beforehand how hard it was gonna be to sleep in a sling [laughter], no idea at all. . . it was a bit of a nightmare’ [P230]*

‘Awful. . . I sorta went home with it on and I just - I just wanted to get it off, I couldn’t stand it.’ [P437]

The movement limitations due to the operation and rehabilitation programme also raised issues:

*‘Because I’m such an active person, the fact that I couldn’t use my arm properly for about six – well, four to six weeks, erm was really demoralising more than anything. Because I couldn’t dress myself, I had to ask my husband to wash me, things that are private to me. . .’ [P260]*

In contrast, most patient participants found early removal of the sling to be tolerable and felt positive about the experience:

*‘No, not a problem at all. . .I think it was the second day after the operation. . .I found it more uncomfortable to be in the sling than to be out of the sling. . .’ [p609].*

‘. . . yeah, I was very happy with it (early removal of the sling), um. . .It allowed me to do it, you know rather than sort of doing it rather sneakily behind the consultant’s back as I’d done previously. Um, yeah, it was good to sort of have the, the choice of I can take it out and have control. . .’ [P449].

Healthcare practitioners also reported favourable feedback from participants randomised to the early patient-directed rehabilitation intervention:

*‘. . .it was nice to see patients get out the sling sooner and get their – get their shoulders moving a little bit sooner. I think it reduced, from what I could tell you know, it reduced fear in the patients.’ [H08].*

Some participants in both groups reported different experiences to the majority. Some reported that participants told them they had little difficulty wearing the sling and some participants found early removal of the sling a challenge:

*‘Like I said, a couple of the patients were – were erm you know, were a little bit reluctant to take their sling off sooner. . .’ [H08]*

‘I found that fairly, you know, straightforward (to wear the sling for four weeks), there were no problems with that. . .’ [P603]

Early removal of the sling was acceptable for most of the physiotherapists taking part. However, one physiotherapist admitted feeling conflicted when delivering the early patient-directed rehabilitation intervention as they were concerned about compromising the surgical repair:

*‘The particular patient that I had erm had had quite extensive surgery. . . And normally with this patient group our surgeons like to be quite conservative, so that part felt a little bit odd to begin with. . .but once. . .the patient was doing well and he was really motivated and was enjoying being able to move their arm and do stuff, then I found that fine’ [H07].*

Some healthcare practitioner participants discussed whether early patient-directed rehabilitation was acceptable for all patients. For some, it was the issue of compromising the surgical repair, yet for others, factors such as age and anxiety were more significant barriers to early removal of the sling:

*‘. . .it obviously depends on the patient. . .I think it might be different if I had a patient that was really anxious. . .’ [HO1].*

‘until you actually see what a rotator cuff looks like, it’s very difficult to understand the nature of that repair. So randomising people outside of the actual intervention becomes technically difficult. . .’ [H09]

There was some evidence of these thoughts of healthcare practitioners, being passed on to patient participants as there were some incidents where patients reported they had been told by healthcare practitioners involved in the trial to be careful, despite the focus of the intervention on being guided by symptoms:

*‘. . . one of my patients did incredibly well, was seen in clinic by a consultant that had signed him up to the trial. And erm he’d said ‘Oh, I was in the gym’,. . .And the surgeon kind of said ‘Oh, you’re too soon to be in the gym, you shouldn’t be doing that’. So that’s kind of the things that we kind of battle with. . .’ [H07]*

‘. . . I was told (by the surgeon) I have to be very very careful if err anything happens like, then err, err they won’t be able to operate again’ [P223]

The recruitment processes used in the pilot and feasibility randomised controlled trial were acceptable to both patient participants and healthcare practitioners:

*‘I just got enough information. If, I needed to speak to anybody I’d speak to them and then get more information. No, the whole process was, was really good’ [P233]*

With regards to data collection, both patient and healthcare practitioner participants found the patient reported outcome measures and responding to text messages acceptable:

*‘It was easy, yeah. . .It was just, you know, asking yes and no or fairly good, good, excellent, stuff like that.’ [P214]*

‘Really, really easy actually. Compared to some of the other studies we work on, really, really easy. Firstly, I think the fact that there’s only a small number of questionnaires selected w-, - is obviously a really good thing for the patient. It makes it much easier’ [H05]

Patient participants and healthcare practitioners did not favour one particular outcome measure, however, there were consistent reports that for some participants, the adherence diary (used to measure time out of sling and adherence to prescribed exercises) was too complicated:

*‘. . .it was a little bit confusing as to you know, how many times you have it out the sling, how many minutes. . .’ [P260]*

The main point of contention for patient participants was the concealment of the research related diagnostic ultrasound scan results. Despite the process being explained as part of the randomised controlled trial consent process, they were unhappy about not having their ultrasound scan results revealed to them:

*‘Somebody’s got to tell you, ‘Your scan’s alright’ or ‘No, there’s a little problem there’. You must tell people that, whether it’s you or the surgeon, but they must tell you. You can’t hide the fact, that’s like telling you you’ve got cancer, but we can’t tell you’ [P241]*

Overall, patient participants and healthcare practitioners were positive about their experiences of being part of the pilot and feasibility randomised controlled trial:

*'Erm, well, the best thing I could say is, I’d take part in another one’ [P406].*

## Discussion

These findings suggest that participants were motivated to enter the randomised controlled trial for the potential to remove their sling early and begin moving their arm. For some, sling use was unacceptable and this contributed to their pain, rather than relieving it, and also compromised self-efficacy. Tensions from some healthcare practitioners, in terms of what the early patient-directed rehabilitation intervention might do to the surgical repair, were apparent and contributed to anxiety about this. Participants found the randomised controlled trial processes, including recruitment, follow-up, and outcome measurement to be largely acceptable, however withholding the results of the ultrasound scan was contentious.

These findings have implications for the design of a future fully powered randomised controlled trial. Where patients express a strong preference for one treatment, as they did in this randomised controlled trial where only 4% of participants expressed a preference for sling use, this risks recruitment of a sample of patients that are not representative of the wider population of patients who undergo surgical repair of the rotator cuff.^10^ Aligned with this is the potential for resentful demoralisation for those patients who consent to participate in the randomised controlled trial due to these perceived benefits of early patient-directed rehabilitation but are then randomly allocated to the standard rehabilitation group.^10^ This risks a biased estimate of clinical outcome via self-report patient-reported outcome measures. Recognising this, the rationale presented for a future fully powered randomised controlled trial would need to be clear and balanced, recognising the current state of uncertainty as well as current understanding of the risks and benefits of each intervention, for example perceived risk of re-tear with early patient-directed rehabilitation, and discomfort and challenge experienced by some with sling use.

The challenge of sling use discussed by some patients was not expected. It is common for slings to be recommended ‘for comfort’ following rotator cuff repair surgery.^11^ Despite this, self-reported time out of sling data in the randomised controlled trial suggested a significant difference in sling use between the early patient-directed rehabilitation and standard rehabilitation groups.^2^ However, in recognition of this finding, and in line with recommendations from our patient and public involvement consultations, it could be advantageous to disclose the challenges faced by some patients to help manage expectations around sling use in the future fully powered randomised controlled trial.

Clinical tension and anxiety about compromising the surgical repair with early patient-directed rehabilitation was apparent. Recent international randomised controlled trials have reported similar re-tear rates in early versus delayed mobilisation groups,^12,13^ but were not powered to detect a difference in re-tear rates between groups. To enable a valid comparison in a future randomised controlled trial this tension needs to be recognised and pro-actively discussed with the aim of working with clinicians in equipoise.^14^

Participants were disgruntled by not being told about their ultrasound scan results. This was a purposeful decision undertaken in consultation with our patient and public involvement group. Reasons include that post-operative imaging is not routinely undertaken in the English National Health Service, and approximately 40% of patients have a re- but still report good quality of life outcomes.^15^ In this situation, where a patient is reporting good outcomes following surgery but is then told that their surgery has failed from a structural perspective, a dilemma is faced. However, given the strength of this feeling, one option for the future randomised controlled trial would be to share ultrasound scan findings with patient participants via their clinical team so that they can be counselled appropriately and supported as necessary.

By nature of the recruitment to the pilot and feasibility randomised controlled trial, the degree to which these findings are transferable to the wider population of surgeons performing rotator cuff repair surgery, physiotherapists supporting post-operative rehabilitation, and patients undergoing rotator cuff repair is uncertain. However, the main aim of this study was to inform the decisions about a future fully powered randomised controlled trial.

The implications of this nested qualitative study include the need to recognise the difficulties some patients face when asked to keep their shoulder sling on for four-weeks following surgery. This finding is relevant to the design of a future trial but also to clinical practice. Furthermore, despite research evidence suggesting benefit of early mobilisation without long-term consequence, it is apparent that the concept of early mobilisation creates tension for some clinicians. Such tension is potentially a challenge to recruitment to a future trial but also a potential barrier to implementation of research evidence and warrants further consideration and discussion between patients, clinicians and researchers to understand and address these concerns.

## Conclusion

These findings suggest that participants were motivated to enter the randomised controlled trial due to the potential for removing their sling and getting moving early. For some participants, sling use was challenging and added to their pain rather than relieving it. Tensions surrounding early patient-directed rehabilitation were apparent in terms of what this might do to the surgical repair and hence lack of equipoise is a concern. Participants found the randomised controlled trial processes largely acceptable; however withholding the results of the ultrasound scan was contentious. These issues will be factored into the design of a future fully powered randomised controlled trial.

**Clinical messages**

- Following rotator cuff repair, many patients want to remove their shoulder sling and start moving.
- Some patients find the sling a hindrance that increases, rather than relieves, their pain.
- Clinicians experience tension about early mobilisation with regard to the effect this might have on the integrity of the surgical repair.

## Supplemental Material

sj-pdf-1-cre-10.1177_0269215520984025 – Supplemental material for Rehabilitation following rotator cuff repair: A nested qualitative study exploring the perceptions and experiences of participants in a randomised controlled trial Click here for additional data file. Supplemental material, sj-pdf-1-cre-10.1177_0269215520984025 for Rehabilitation following rotator cuff repair: A nested qualitative study exploring the perceptions and experiences of participants in a randomised controlled trial by Gareth Stephens, Chris Littlewood, Nadine E Foster and Lisa Dikomitis in Clinical Rehabilitation

sj-pdf-2-cre-10.1177_0269215520984025 – Supplemental material for Rehabilitation following rotator cuff repair: A nested qualitative study exploring the perceptions and experiences of participants in a randomised controlled trial Click here for additional data file. Supplemental material, sj-pdf-2-cre-10.1177_0269215520984025 for Rehabilitation following rotator cuff repair: A nested qualitative study exploring the perceptions and experiences of participants in a randomised controlled trial by Gareth Stephens, Chris Littlewood, Nadine E Foster and Lisa Dikomitis in Clinical Rehabilitation

sj-pdf-3-cre-10.1177_0269215520984025 – Supplemental material for Rehabilitation following rotator cuff repair: A nested qualitative study exploring the perceptions and experiences of participants in a randomised controlled trial Click here for additional data file. Supplemental material, sj-pdf-3-cre-10.1177_0269215520984025 for Rehabilitation following rotator cuff repair: A nested qualitative study exploring the perceptions and experiences of participants in a randomised controlled trial by Gareth Stephens, Chris Littlewood, Nadine E Foster and Lisa Dikomitis in Clinical Rehabilitation
