# Supplementary material for: Rehabilitation following rotator cuff repair: A nested qualitative study exploring the perceptions and experiences of participants in a randomised controlled trial
Source: Clin Rehabil. 2020 Dec 27;35(6):911–9. doi: 10.1177/0269215520984025 (PMC8191163; doi:10.1177/0269215520984025)
Supplement: sj-pdf-1-cre-10.1177_0269215520984025 – Supplemental material for Rehabilitation following rotator cuff repair: A nested qualitative study exploring the perceptions and experiences of participants in a randomised controlled trial [file sj-pdf-1-cre-10.1177_0269215520984025.pdf]

Appendix One: Patient participant topic guide

1. Introductions

- Age, Employment, Role
- Activity levels, Hobbies

2. Understanding

- This is not a test we are asking everyone this question out of interest…..
- Can you explain the study to me in your own words….

3. Journey prior to surgery (brief)

- How it started?
- When it started
- Prev treatments / medications

4. Role in research

- Which group are you in?
  - How did you feel about this when you were told?
  - Comfortable in the sling / removing the sling.

5. Recruitment

- Experience of being approached
- First phone call
- Study documents
- Being approached in POAC - ? time to ask questions
- Changes?

6. Data collection

- Questionnaire at POAC
- Exercise diary
- Second questionnaire at 6 weeks – post
- Weekly text messages?
- Did you feel the questions captured your experience or would you like to have seen something added?
- 12-weeks USS – experiences / apt time / attendance

7. Experience of treatment

- What was explained to you about why you were having the surgery?
- Experience of having the surgery
  - Relationship with the surgeon?
- Describe your experience of the treatment you received.
- What kind of advice did you receive from physio – inpt and OPD
- Can you describe the kinds of exercises you were asked to do?
- Challenges?
- Experience using / not using sling
- Any concerns?
- Overall negative / positive experiences

8. Wrap up

- Are you able to summarise your overall experience of being part of the trial?
- Do you have any messages you would like me to feed back to the study team that we haven't already discussed?
