# Supplementary material for: Rehabilitation following rotator cuff repair: A nested qualitative study exploring the perceptions and experiences of participants in a randomised controlled trial
Source: Clin Rehabil. 2020 Dec 27;35(6):911–9. doi: 10.1177/0269215520984025 (PMC8191163; doi:10.1177/0269215520984025)
Supplement: sj-pdf-2-cre-10.1177_0269215520984025 – Supplemental material for Rehabilitation following rotator cuff repair: A nested qualitative study exploring the perceptions and experiences of participants in a randomised controlled trial [file sj-pdf-2-cre-10.1177_0269215520984025.pdf]

Appendix Two: Healthcare practitioner involved in recruitment topic guide

1. Introductions

- Age, Year in role, Spec interests
- Previous clinical trials?

2. Understanding

- This is not a test we are asking everyone this question out of interest…..
- Can you explain the study to me in your own words….

3. Screening

- How easy was it to identify eligible patients?
- Anything different?

4. First contact with patient

- In general, how did the phone calls go?
- Describe any positive or negative experiences.
- Patient reaction to phone calls?
- Was the level of information? Well understood?
- What were the most difficult issues to explain over the phone?
- Anything different?

5. Consent in point of access clinic

- In general, how easy was it to arrange the appointments and go through the consent in POAC?
- What challenges did you face?
- Did you face any recurring questions from patients?
- Were there any issues you found difficult to explain?
- Did you miss any patients? Reasons?
- Lessons learned.

6. Data collection

- Process of collecting baseline questionnaire?
- Did you face any recurring questions from patients?
- Any strange / most memorable requests…..
- Lessons learned? / Improvements?

7. Randomisation

- What were your experiences in accessing the online randomisation service?
- Any reflections on the process of concealing the allocation in the envelope?
- Any improvements?

8. Wrap up

- Are you able to summarise your overall experience of being part of the trial?
- Do you have any messages you would like me to feed back to the study team that we haven't already discussed?
