# Supplementary material for: Rehabilitation following rotator cuff repair: A nested qualitative study exploring the perceptions and experiences of participants in a randomised controlled trial
Source: Clin Rehabil. 2020 Dec 27;35(6):911–9. doi: 10.1177/0269215520984025 (PMC8191163; doi:10.1177/0269215520984025)
Supplement: sj-pdf-3-cre-10.1177_0269215520984025 – Supplemental material for Rehabilitation following rotator cuff repair: A nested qualitative study exploring the perceptions and experiences of participants in a randomised controlled trial [file sj-pdf-3-cre-10.1177_0269215520984025.pdf]

Appendix Three: Physiotherapists’ topic guide

1. Introductions

- Age
- Year in role
- Spec interests
- Previous clinical trials?

2. Role in research
- In patient or out patient

3. Understanding
- This is not a test we are asking everyone this question out of interest…..
- Can you explain the study to me in your own words….

4. Training
- Describe experiences of training
- Confidence?
- Any different?
- Session content / logistics?
- How would you feel if training was online?

5. Intervention delivery
- Did you treat patients in both the early mob and standard care groups?
- Describe your **experiences of treating early mob** group
    - Confidence to deliver / niggling doubts
- Describe your **experiences of treating standard care** group
- Were you aware of **tear size / location?** Did this affect how you delivered the intervention?
- Personal exercise diaries –
    - competed?
    - were you able to support completion?
    - did they help you monitor progress?
    - compliance?
    - Returned?
    - Any changes
- What went well for each group
- What challenges did you face?

6. Wrap up
- Are you able to summarise your overall experience of being part of the trial?
- Do you have any messages you would like me to feed back to the study team that we haven’t already discussed?
